# A scoping review on the clinical effectiveness of Trans-Impedance Matrix (TIM) measurements in detecting extracochlear electrodes and tip fold overs in Cochlear Ltd devices

**DOI:** 10.1371/journal.pone.0299597

**Published:** 2024-03-07

**Authors:** Muhammed Ayas, Jameel Muzaffar, Daniele Borsetto, Susan Eitutis, Veronica Phillips, Yu Chuen Tam, Marina Salorio-Corbetto, Manohar L. Bance

**Affiliations:** 1 College of Health Sciences, University of Sharjah, Sharjah, United Arab Emirates; 2 Emmeline Centre, Cambridge University Hospitals NHS Foundation Trust, Cambridge, United Kingdom; 3 Cambridge Hearing Group, University of Cambridge, Cambridge, United Kingdom; 4 Department of Ear, Nose and Throat Surgery, University Hospitals Birmingham NHS Foundation Trust, Birmingham, United Kingdom; 5 Department of ENT, Cambridge University Hospitals NHS Foundation Trust, Cambridge, United Kingdom; 6 Medical Library, University of Cambridge School of Clinical Medicine, Cambridge, United Kingdom; University of Szeged: Szegedi Tudomanyegyetem, HUNGARY

## Abstract

**Background:**

Extrusion of electrodes outside the cochlea and tip fold overs may lead to suboptimal outcomes in cochlear implant (CI) recipients. Intraoperative measures such as Trans-Impedance Matrix (TIM) measurements may enable clinicians to identify electrode malposition and direct surgeons to correctly place the electrode array during surgery.

**Objectives:**

To assess the current literature on the effectiveness of TIM measurements in identifying extracochlear electrodes and tip fold overs.

**Methods:**

A scoping review of studies on TIM-based measurements were carried out using the Databases-Medline/PubMed, AMED, EMBASE, CINAHL and the Cochrane Library following PRISMA guidelines. Eleven full texts articles met the inclusion criteria. Only human studies pertaining to TIM as a tool used in CI were included in the review. Further, patient characteristics, electrode design, and TIM measurement outcomes were reported.

**Results:**

TIM measurements were available for 550 implanted ears with the subjects age ranged between 9 months to 89 years. Abnormal TIM measurements were reported for 6.55% (36). Tip fold over was detected in 3.64% (20) of the cases, extracochlear electrodes in 1.45% (8), and 1.45% (8) were reported as buckling. Slim-modiolar electrode array designs were more common (54.71%) than pre-curved (23.34%) or lateral wall (21.95%) electrode array. Abnormal cochlear anatomy was reported for five ears (0.89%), with normal cochlear anatomy for all other patients.

**Conclusion:**

TIM measurement is a promising tool for the intraoperative detection of electrode malposition. TIM measurement has a potential to replace intraoperative imaging in future. Though, TIM measurement is in its early stages of clinical utility, intuitive normative data sets coupled with standardised criteria for detection of abnormal electrode positioning would enhance its sensitivity.

## Introduction

Cochlear implants (CIs) are devices that are surgically implanted into the inner ear of individuals with severe to profound hearing loss [1]. CIs have been shown to be effective in improving speech understanding, sound localization, and overall quality of life [2–4]. However, CI outcomes are variable, and results can depend on the effectiveness of the surgical placement of electrodes in the scala tympani, thereby optimising use of the available information channels through the implanted electrodes. There may be significant reduction in CI performance when electrodes migrate, or are partially inserted at the time of surgery [5–7]. Electrodes outside the cochlea are termed extracochlear electrodes (EE).

The goal of minimizing the electrode-neural gap for lower power use and theoretically more focused stimulation of the auditory nerve, has prompted the use of pre curved, peri-modiolar (e.g. Slim Modiolar Electrode-SME^®^) type electrode arrays for CI surgery, which could result in better hearing performance in CI recipients [8,9]. However, a problem encountered during electrode positioning is that of Tip Fold Over (TFO). Both TFO and EE can adversely impact the effectiveness of the CI [10,11]. TFO can result in a shorter active length of the electrode array which can lead to reduced stimulation of the auditory nerve and the current spread from the apical electrodes to the unintended electrodes create cross turn stimulation. Additionally, there is a possibility that EE may still stimulate basal regions of the cochlea that are already activated by intracochlear electrodes, resulting in multiple electrodes stimulating the same neural region [11]. This can negatively affect speech understanding reducing the overall benefit from the implant, and in some cases may even require revision surgery [12–14]. It is therefore important to understand and address this problem in order to optimize the effectiveness of CI.

Electrode impedance measures (from the active electrodes to the return electrode placed on the CI case or ring/lead ground) during and after CI surgery are the most commonly used tests to probe the status of electrode arrays inside the cochlea. However, a finding of normal electrode impedance measure may not be an indication of electrode misplacement in the cochlea [13,15]. One recent development in the field of CI clinical research is the introduction of Trans-Impedance Matrix (TIM) measures for the measurement of the electrode voltage profile, which subsequently helps in identification of TFO or EE. These are termed Trans-Impedance Matrix (TIM) by Cochlear^®^ Ltd, Electric Field Imaging (EFI) by Advanced Bionics^®^, Impedance Field Telemetry (IFT) by MED-EL^®^, and more generally, Stimulating Current Induced Non-Stimulating Electrode Voltages (SCINSEVs) [11]. As the body of literature is for the Cochlear Ltd devices^®^, the term TIM will be used in what follows. TIM is measured by applying a small current to each electrode and measuring the resulting induced voltage across all other non-stimulating electrodes. This results in a 22 by 22 matrix, which can be plotted as a graph or used to create a heat map representing the voltage matrix of the electrode array [11]. In addition to intraoperative testing, TIM can be used to monitor the status of electrodes postoperatively. This can be useful for detecting changes in the electrical properties of electrodes due to factors such as tissue growth or scarring, which can also affect the current delivery from the CI [16,17]. The increased interest in the clinical use of TIM is partly due to the ease with which the test can be completed, without any additional support/equipment added to the clinical/intraoperative testing set up.

There has been an increase in TIM research over the past few years. Therefore, it is important to understand the use of TIM intraoperatively and postoperatively to evaluate the status of the electrode array [18,19]. Synthesizing this evidence allows for more informed use of these measures, and wider adoption of TIM testing in CI recipients. To the best of our knowledge, there have been no reviews reported on the effectiveness of TIM in identifying TFO and EE, or the criteria used to make these judgements. This is the purpose of this review. Further, we aim to determine what has already been done and where gaps and opportunities to be addressed by future research are. The objective was to catalogue current research on TIM as a tool used intraoperatively, postoperatively, characterise the population that has been studied, determine the type of electrode arrays that have been used and what the clinical implications of using TIM are.

## Methods

We conducted this scoping review broadly based on the methodological framework outlined by Levac and colleagues [20], which involves five key stages 1) identifying the research questions (above); 2) identifying relevant studies; 3) selecting studies; 4) charting the data; and 5) collating, summarising and reporting results, as well as based on the guidelines outlined in the preferred reporting items for systematic reviews and meta-analyses extension for scoping reviews (PRISMA-ScR) [21]. The scoping review was prospectively registered on the Open Science Framework (OSF) database on December-12-2022 (https://doi.org/10.17605/OSF.IO/P6ZTX).

### Identifying relevant studies

The databases Medline (via Ovid), Embase (via Ovid), Cochrane Library, Scopus, and Web of Science (Core Collection) were searched by a Clinical Librarian (VP) from inception to March 2023. Results were filtered using the English language limit on all databases. The search strategy was peer-reviewed by two librarian colleagues of VP using the PRESS checklist and evaluated against the PRISMA-ScR guidelines (See S1 File). Databases were searched separately, rather than multiple databases being searched simultaneously on the same platform to improve accuracy. The search syntax was adapted for each database, and to account for variation between thesaurus terms/controlled vocabulary across each database. Results were deduplicated using Endnote 20 software.

### Study selection

The literature search was carried out by outlining the PIO. Children and Adults with CI as the population (P) of interest. The studies focussed on measuring TIM intraoperatively and postoperatively to detect EE and TFO served as intervention (I) and outcome (O) measures, respectively. Studies reporting EFI and IFT profiles (from Advanced Bionics and MED-EL) have not been included in the review. No restrictions were placed on study design for the inclusion. Studies carried out as cross-sectional studies, longitudinal, experimental, quasi experimental and observational studies were all included in the review. Case reports, editorials and studies not conducted in English or lacking an English translation were excluded from the review.

All articles before March 2023 were considered, including published and in press articles. Studies conducted as part of translational research which involved both human and cadaveric data were included by extracting the human data. Animal studies or those using temporal bone data only were excluded.

The outline search key words (See S2 File) were entered into the search database as free text as well as controlled texts. By applying the selection criteria, title screening was performed independently by two authors (MA and JM). Abstracts of the included titles were screened and full text articles were shortlisted and extracted. The reference lists of all full text articles were screened in order to identify any studies not highlighted by database searches. Discrepancies raised during the process of article screening such as variations in recorded data and challenges in differentiating study designs, were discussed, and resolved through consensus by a third author (MB). Finally, articles which met the inclusion criteria were presented for full text review and data extraction.

### Charting the data

A data extraction form was developed by the review team and used for the data extraction. Each study was independently reviewed for data extraction by two authors (MA, JM), with any discrepancies resolved by discussion. Data extraction was focused under the following domains: author details, study design, study sample, age range, type of CI electrodes used, imaging status of cochlea and TIM results. Additionally, key research questions from all included studies and their key findings were extracted and listed.

#### Quality appraisal of the studies

Studies were assessed for quality and risk of bias by the reviewers according to the Cochrane Handbook for Systematic Reviews of Interventions [22]. The quality of the studies included for review was assessed using the Downs and Black Checklist for Measuring Quality [23] to evaluate the study quality, external validity, bias of the study, the confounding and selection bias, as well as the power of the study (Table 1). No conflict of interest or financial incentives were declared in any of the studies used within the review. Discussion and consensus were reached between the authors when there was a discrepancy in the ratings assigned to studies.

**Table 1:** Black and Downs table showing internal and external validity, bias and reporting for studies included for review.

	Klabbers et al (2021)	Soderqvist et al (2021)	Hans et al (2021)	Ramos-de-Miguel et al (2021)	Klabbers at al (2021)	Hoppe et al (2022)	Vozzi et al (2022)	Leblans et al (2022)	de Rijk et al (2022)	Kay riverst et al (2022)	Cheung et al (2022)
**REPORTING**											
Q1.Hypothesis/objective clearly described	1	1	1	1	1	1	1	1	1	1	1
Q2.Main outcomes in Introduction or Methods	1	1	1	1	1	1	1	1	1	1	1
Q3.Patient characteristics clearly described	1	1	1	1	1	1	1	1	1	1	0
Q4.Interventions of interest clearly described	1	1	1	1	1	1	1	1	1	1	1
Q5.Principal confounders clearly described	1	1	1	1	1	1	1	1	1	1	0
Q6.Main findings clearly described	1	1	1	1	1	1	1	1	1	1	1
Q7.Estimates of random variability provided for main outcomes	1	1	1	1	1	1	1	1	1	1	1
Q8.All adverse events of intervention reported	1	1	1	1	1	1	1	1	1	1	1
Q9.Characteristics of patients lost to follow-up described	1	UTD	1	1	UTD	1	1	1	UTD	1	1
Q10.Probability values reported for main outcomes	1	1	1	1	1	1	1	1	1	1	1
**EXTERNAL VALIDITY**											
Q11.Subjects asked to participate were representative of source population	1	1	1	1	1	1	1	1	1	1	1
Q12.Subjects prepared to participate were representative of study population	1	1	1	1	1	1	1	1	1	1	1
Q13.Location and delivery of study treatment was representative of source population	1	1	1	1	1	1	1	1	1	1	1
**INTERNAL VALIDITY–BIAS & CONFOUNDING**											
Q14.Study participants blinded to treatment	0	0	0	0	0	0	0	0	0	0	0
Q15.Blinded outcome assessment	0	0	0	0	0	0	0	0	0	0	0
Q16.Any data dredging clearly described	0	0	0	0	0	0	0	0	0	0	0
Q17.Analyses adjust for differing lengths of follow-up	1	1	1	1	1	1	0	1	0	0	1
Q18.Appropriate statistical tests performed	1	1	1	1	1	1	1	1	1	1	1
Q19.Compliance with interventions was reliable	1	1	1	1	1	1	1	1	1	1	1
Q20.Outcome measures were reliable and valid	1	1	1	1	1	1	1	1	1	1	1
Q21.All participants recruited from the same source population	1	1	1	1	1	1	1	1	1	1	1
Q22.All participants recruited over the same time period	1	1	1	1	1	1	1	1	1	1	1
Q23.Participants randomised to treatment(s)	0	0	0	UTD	0	0	UTD	0	0	0	1
Q24.Allocation of treatment concealed from investigators and participants	0	0	0	0	0	0	0	0	0	0	0
Q25.Adequate adjustments for confounding	0	0	0	0	0	1	0	1	0	1	0
Q26.Losses to follow-up taken into account	0	UTD	1	1	UTD	1	1	1	1	1	0
**POWER**											
Q27.Sufficient power to detect treatment effect at significance level of 0.05	0	0	1	0	0	1	1	1	0	1	0
**TOTAL**	**19**	**18**	**21**	**20**	**18**	**21**	**20**	**22**	**18**	**21**	**18**

UTD- Unable to determine; Scoring: 0(No) or 1(yes).

## Results

### Source of evidence

**Fig 1:**
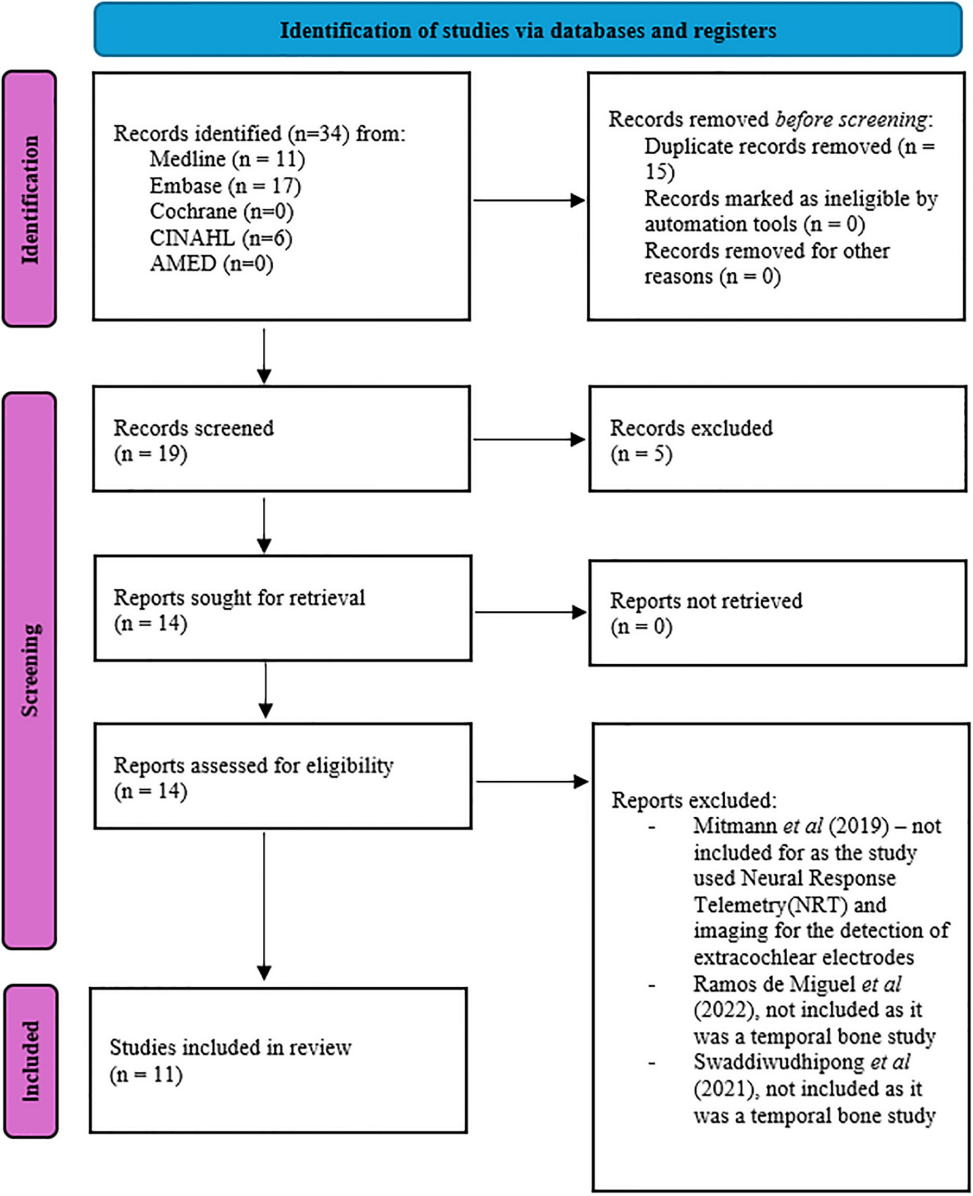
PRISMA flowchart for study identification and selection.

A total of 34 studies were identified through the search. Of these, 15 duplicated records were excluded. Abstracts for the 19 remaining articles were screened and five were excluded. Finally, 14 full-text records were thoroughly assessed by the reviewers and three studies were excluded. The remaining 11 full-text articles were included in the review and the data were extracted. The screening, selection, and inclusion processes were illustrated in the PRISMA chart (Fig 1).

### Characteristics of the sources of evidence

Studies were conducted in North America-the United States [33], Europe- including the United Kingdom [32], Netherlands [24,28], Germany [26], Finland [25], Italy [30], Spain [27], Belgium [31], Asia Pacific-Australia [34]. One study was an international multicentre collaboration (Australia, Germany, and Spain) [29]. No reported studies were found from South America or from Middle East and Africa. It is noteworthy that all published studies included in this scoping review were conducted between 2021 to 2022. It is important to highlight that no date restrictions were applied to the initial search strategy, thus supplementing the novelty and relevance of the studies included in this review.

### Results from the individual source of evidence

Table 2 summarizes the individual characteristics of the included studies. Table 3, summarizes the key research questions main findings for each study.

**Table 2:** Characteristics and sampling of the study population.

Study [reference citation]	Sample	Study Design	Age	Electrode Design			Imaging	Cochlear Anatomy		TIM				Imaging	
	Size		(Mean)												
				CI 512/612	CI	CI	Pr-OP	Normal	Abnormal	Normal	TFO	EE	Buckling	I-OP	P-OP
					522/622	532/632									
**Klabbers et al., 2021** [24]	25	Proof of Concept	NA	NIL	NIL	CI 532,632	Yes	23	2	22	3	0	0	X-ray	CT
**Soderqvist et al., 2021** [25]	51	Retrospective	31	NIL	CI522,CI622	NIL	Yes	51	0	51	0	0	0	No	CT
**Hans et al.,2021** [26]	100	Retrospective	NA	CI 512/612	CI 522/622	CI 532/632	Yes	100	0	95	4	0	1	X-ray	No
**Ramos-de-Miguel et al.,2021** [27]	24	observational	41.3	NIL	CI 622	CI 632	Yes	24	0	24	0	0	0	No	CB-CT
**Klabbers et al., 2021** [28]	47	Retrospective	NA	NIL	NIL	CI 632	Yes	47	0	44	3	0	0	X-ray	No
**Hoppe et al.,2022** [29]	148	Prospective observational	58	CI 512	NIL	CI 532	Yes	148	0	144	4	0	0	CT/CB-CT	CT/CB-CT
**Vozzi et al.,2022** [30]	24	Prospective	44	CI 512	NIL	CI 532/632	Yes	44	0	NA	NA	NA	NA	No	CT
				CI 24RE*											
**Leblans et al.,2022** [31]	20	Prospective longitudinal	56.6	CI 512	NIL	CI 532	Yes	20	0	20	0	0	0	No	CT/CB-CT
**de Rijk et al.,2022** [32]	6	Retrospective	NA	CI 512	CI 522	NIL	Yes	NA	NA	0	0	6	0	No	X-ray
**Kayrivest et al.,2022** [33]	117	Retrospective	59.9	CI 612	CI 622	CI 632	yes	114	3	112	3	2	0	X-ray	No
					/CI 624**										
**Cheung et al.,2022** [34]	12	Retrospective	NA	CI 512	CI622	CI 532	Yes	NA	NA	2	3	0	7	X-ray	X-ray

**CI**-Cochlear Implant; **EE**-extracochlear electrodes; **I-OP**-Intra-Operative; **NA**-Not Available; **NIL**- not used specific electrode design;

*Peri-modiolar electrodes; **Pr-OP**-Pre-Operative; **P-OP**-Post-Operative

**Slim 20 electrodes; **TIM**-Tran-impedance Matrix; **TFO**-Tip fold over.

**Table 3:** Research questions and key findings.

Study [reference citation]	Key research Question	Summary of main findings/results
**Klabbers et al., 2021** [24]	To investigate the use of intraoperative TIM in detection of TFO compared to intraoperative imaging in a specific electrode array- SME	Electrode TFO was reported in three of the 25 CIs (12%) based on TIM along with imaging fluoroscopy intraoperatively, suggesting that TIM is an easy, fast and valuable tool in replacing imaging requirements intraoperatively for the detection of TFO with SME array.
**Soderqvist et al., 2021** [25]	To study the spread of intracochlear electrical field using TIM and SOE measures using LW electrode array	There was a strong correlation between SOE and TIM in the intracochlear electrical field for LW electrode array, dominantly on the apical array. Further analysis of the results indicates there is a concentrated effect of TIM and SOE seen on larger cochlear size. However no intraoperative imaging was done after TIM. Electrode positioning was further verified using CT scans postoperatively,
**Hans et al., 2021** [26]	To study the accuracy of TIM profiles with that of SOE and imaging for the prediction of electrode malposition in the cochlea.	7% of the patients (7/100) had abnormal TIMs. The sensitivity (97.8%) and specificity (100%) were high in detection of TFO compared to SOE. All TFO were detected accurately by TIM and intraoperative x-ray without any false negative finidings. TIM heat maps provide useful information about the location and positioning of the electrode array in cochlea.
**Ramos-de-Miguel et al., 2021** [27]	To understand the effect of SME position and stimulation using TIM and radiological measures.	TIM heat maps showed significant intersubjective variability. SME heat map were more stable than LW heat maps. Electrode array position was confirmed postoperatively with CBCT and no abnormalities were reported. Further, the study reports the impedance increases as the distance from the modiolus increases
**Klabbers et al., 2021** [28]	To compare the TIM and X-ray fluoroscopy for the intraoperative detection of TFO	6.4% (3/47) of cases with TFO were detected. Inter-rater agreement was 88% (Cohens k = 0.378) for intraoperative fluoroscopy and 99% (Cohens k = 0.915) for TIM. The inter-rater agreement for TIM measurement was reported ‘‘near perfect” (Fleiss’ K = 0.850). TIM could potentially replace intraoperative imaging in the future.
**Hoppe et al., 2022** [29]	To study the specificity of an algorithm for the detection of TFO and electrode position inside cochlea using LW and SME array.	Four cases of TFO were detected out of 148 ears implanted (2.70%). CT imaging were performed intraoperatively or postoperatively depending on the clinic protocol of the study centres. No TFO was detected in CT imaging. Roll off of TIM is rapid on the basal end compared to the apical end. The TIM algorithm has a specificity and sensitivity of more than 95% for the detection of intracochlear electrode anomalies.
**Vozzi et al.,2022** [30]	To study the innovative use of TIM in specifying hearing pathologies based on TIM quantitative analysis	Two groups of patients (congenital and otosclerosis) were studied with three indices: 1) Shannon entropy; 2) Exponential decay are used to study the electrical field properties in the congenital hearing loss; and 3) Spatial correlation was used for measuring the homogeneity of TIM in the hearing pathologies. The three indices differed across basal and apical electrodes. Postoperative CT imaging showed normal placement of electrode array in all subjects. Further, spatial correlation showed TIM has distinct pattern based on hearing pathologies. Findings support the use of quantitative analyses of TIMs to detect hearing pathologies.
**Leblans et al.,2022** [31]	To study the effectiveness of EVT/TIM as a biomarker for postoperative fibrous tissue formation in CI	TIM indicated no indication of TFO, which was confirmed postoperative CT/CBCT imaging Further the study demonstrates the time scale of postoperative tissue growth by monitoring the access changing resistance values. The study also highlights the changes in far filed resistance during and after CI, indicating the flow of current near the round window will be further inhibited due to fibrous tissue growth postoperatively.
**de Rijk et al.,2022** [32]	To study the detection of extracochlear electrodes using in SCINSEVs	An algorithm for the detection of EE was used, which had a sensitivity and specificity of 91% in cadaver specimens was used to analyse intraoperative recordings obtained in 06 humans. Postoperative x-ray was used to verify the electrode positioning. Quantification of EE between estimated and actual EE showed a good correlation (r = 0.69). Changes were observed in the SCINSEV pattern in the transition zone from intracochlear to extracochlear electrodes in all cases. Further, SCINSEV finding were compared with surgeon’s report on insertion. However, some mismatch was found which possibly indicate the postoperative migration of electrodes or error in the quantification by the surgeon or by the algorithm.
**Kayrivest et al.,2022** [33]	To investigate the TIMs reliability to detect TFO compared to the gold standard intraoperative plain film radiograph.	TFO was detected in 2.4% of the cases (3/117) which was confirmed by intraoperative x-ray. The sensitivity and specificity of TIM measurements in detecting electrode TFO were both 100%. In cases of over insertion, TIM appears to be normal but the X-ray was decisive in proving over insertion.
**Cheung et al.,2022** [34]	To create a quick reference guide for the detection of electrode misplacement in the cochlea	Sixteen cases of CI misplacement were reported. A combination of EP, TIM and X-ray (intraoperative and postoperative) were helpful for the detection of the electrode misplacement such as TFO,EE and buckling. Authors’ experience-based quick reference guide was provided for the detection of electrode array misplacement.

**CI**-Cochlear Implant; CBCT- Cone-Beam computed tomography; CT- Computed Tomography; **EE**-extracochlear electrodes; **EVT**-Electrode Voltage Telemetry; **LW**-Lateral Wall; **SCINSEV-** Stimulation-Current-Induced Non-Stimulating Electrode Voltage Recordings; **SME**-Slim Modiolar Electrode; **SOE**: Spread of Excitation; **TFO**-Tip fold over; **TIM**-Tran-impedance Matrix.

### Synthesis of results

A total of 574 CI implanted ears underwent TIM measurements intraoperatively and postoperatively. One study [33] categorized adults and paediatrics separately, whilst five studies included adult and paediatric patients together [25,27,29–31]. Five studies did not mention the mean age range of their data set [24,26,28,31,34]. Excluding these studies, patients had an age range between 9 months and 89 years. The implanted electrode arrays were the profile and profile plus series electrode arrays from Cochlear Ltd.^®^, Sydney, Australia (Cochlear Nuclues-CI-512/612, CI-522/622 and CI-532/632) as well as CI24RE- perimodiolar electrode array. Additionally, one subject received the Slim 20-CI 624 electrode array.

Out of the 574 implanted ears, 24 ears did not have TIM results reported [30]. Considering the remaining 550 implanted ears, there were 36 cases of abnormal TIM (6.55%). This includes detection of TFO on 20 ears (3.65%), EE on 8 ears (1.45%), and buckling of the electrode array on 8 implanted ears (1.45%).

All the studies reported imaging as their standard procedure intraoperative or post implantation to check the electrode status. All but two studies reported patients having normal cochlear structure preoperatively, with the two other studies reporting five abnormally structured cochleae based on CT scans [24,33]. Additionally, two studies [25,26] also included Spread of Excitation (SOE) measures to complement the TIM responses obtained during the test procedure.

Only three studies were prospective longitudinal [27,29,31], by measuring TIM intraoperative and at postoperatively (01,03,06 and 12 months) to predict CI outcome as a function of electrode array position. Six studies were retrospective in nature [25,26,28,32,33,34]. One [24] was a proof-of-concept study examining the potential use of TIMs in intraoperative measurements [30], and one study [29] was a prospective multicentre study designed for the detection of TFO.

## Discussion

TIM utilizes electrode voltage measurements to provide a visual representation of a voltage distribution in the cochlea. As a non-invasive method, TIM is potentially an important tool for the detection of TFO and EE. In this scoping review, we aimed to examine the current state of knowledge on the use of TIM in CI recipients and to evaluate the evidence on its clinical effectiveness. The majority of the studies included in this scoping review arise from a small number of centres, predominantly in Western Europe.

### TIM in detection of TFO

One of the most significant clinical applications of TIM in CI is the detection of TFO, which can lead to decreased performance and reduced hearing ability in CI recipients [24]. Five studies in the review demonstrate that TFO tests demonstrate 100% sensitivity and specificity when compared to intraoperative imaging [24,26,28,33,34]. The findings denote that TIM measurements can effectively detect TFO and offer advantages over the current gold standard of radiological imaging. The benefits include less imaging requirement during the surgery, reducing radiation exposure due to imaging and making the surgical turn around quicker in operating theatres [24].

The heat map shows higher trans-impedances measured at apical contacts compared to basal contacts. Therefore, TIM measurements have the potential to replace intraoperative fluoroscopy as a modality for intraoperative detection of TFO. TFO are particularly seen in SME arrays. Compared to radiological imaging, TIM are easier to perform, less time-consuming and leads to a reduction in surgery time and cost. Although, the current evidence suggests high sensitivity and specificity of TIM measurements, the use of TIM will continue to depend on the expertise of the clinicians intraoperatively in detecting TFO [27].

While the performance of TIM is impressive, measures of accuracy with more certainty requires larger numbers, particularly considering the low incidence of TFO reported. In one of the reviewed studies attempts were made to improve the specificity of TFO detection in the TIM algorithm to assess the electrode array position [29]. Typically, the TIM algorithm calculates the two-dimensional gradients of the matrix by finding out the size and direction. In normally placed electrode arrays, the phase gradient points towards the main diagonal of the matrix with the trans impedance values reducing as it moves away from the stimulating electrode. However, in TFO, the phase gradients are disrupted and points towards different directions. Thus by analysing the range of phase gradient, the algorithm can effectively differentiate TFO from that of normal TIM measurements. The authors of the study suggest that a specificity of 98.6% results with a positive predictive value of 76%, meaning that 3 out of 4 cases flagged as a TFO would be correctly identified [29]. Thus, it is feasible for this type of algorithm to enable the TIM measurements to be easily applied in the operating theatres and therefore used as a routine clinical tool. The overall prevalence of TFO found in their study was 2.5% and the prevalence of TFO in the SME array was 3.9%. However, the study failed to report the sensitivity of the algorithm within their sample. Such an algorithm would enable less experienced clinicians to predict TFO in a more intuitive manner intraoperatively. Once such algorithm for the automatic detection of TFO is available within the Cochlear Nucleus^®^ SmartNav software, which has been recently released by Cochlear^®^ Ltd.

### TIM in detection of EE and electrode position

In addition to detecting TFO, TIM has also been used to detect EE, where an electrode array has migrated out of the cochlea and into the surrounding tissue or the array has not been completely inserted at the time of surgery, which can lead to decreased performance and reduced hearing ability in CI recipients. Studies included in this review suggest that TIM can be used to accurately identify EE, which would help clinicians to make informed clinical decisions during surgery, such as electrode repositioning, reducing the need for revision surgery.

EE and electrode migration are under reported in the literature [13,14,35]. EEs are estimated to be present in between 9.2 to 13.4% of CI recipients, and can cause issues such as non-auditory stimulation and pain when these electrodes are left activated [14,33,36]. Therefore, TIM being an indirect measure of electrical spread along the electrode array would allow early detection of EE [32]. This work was previously completed in cadavers using EFI from Advanced Bionics^®^, which also indicated potential of using indirect measurements of electrical spread for early detection of EE [11].

Another important aspect of TIM in electrode malposition is exploring the width of the TIM profiles, that is by quantifying the spread of electrical field at the stimulating electrodes in the cochlea. It has been reported that width could vary in different parts of the electrode array in the cochlea [25]. The authors of the study reported, when comparing the TIM profile to that of spread of excitation (SOE) profile, the largest differences were seen in the basal end electrodes with TIM being narrower than SOE profiles. While the apical and middle portions showed wider TIM profile similar to that of SOE. It is unclear what causes the discrepancy between the TIM and SOE profiles, however, it is assumed that the poor tissue conductivity around the round window may be causing faster decay of the electrical field from the cochlea [25]. Accurate knowledge of the exact position of intracochlear electrodes is crucial to substantiate such assumptions. Findings from this study indicate that the 50% peak width of both TIM and SOE correlated moderately in the basal part. This has some important implications on locating the EE using TIM. However, factors such as stimulation paradigm used in SOE may affect the comparison of TIM and SOE profiles. Newly developed panoramic electrical compound action potentials (PECAP) method could well be used to overcome those limitations [37]. Further, it might be useful to explore specifically with an electrode-anatomical model based on post-operative CT scans which might help to understand these profiles on an individual level.

It is noteworthy that the electrode voltage profile may vary across electrode arrays and differ in higher cut off values (range: from 0.74 to 2.32) [11]. The optimal cut off value for electrodes differ for three CI manufactures. Cochlear^®^ Ltd, Advanced Bionics^®^, MED-EL^®^ have cut off value of 0.81,1.53 and 2.02 respectively. This has an important clinical value when using TIM compared to other trans impedance measures (EFI, VM) to understand the trend of the voltage matrix with differing electrode designs. Cadaveric experiments have shown that the reported changes can be attributed to the difference in electrode contact spacing between arrays [11,32]. Therefore, lesser contact spacing in the array would produce less abrupt transitions in electrode voltage as seen in TIM measures compared to other voltage measures such as EFI and IFT [32]. Overall, with experience many audiologists and surgeons may find it easier to detect EEs visually with TIM.

In general, TIM is found to be useful in both lateral wall and SME arrays [26,27,30,32,34]. Fewer studies have explored whether TIM recording could provide information about the peri-modiolar vs lateral position of the electrode array [26,27] and its relation to electrode performance, with only one study designed to evaluate the listening effort of peri-modiolar vs lateral wall electrodes in an electrode discrimination test [27].

One study reported [27] that TIM changes correlate with the position of the SME array to the modiolar wall. However, other factors such as conductivity and shape of the cochlea can also affect the TIM decay. If these parameters are not taken to account then, the change in TIM profile only reflect proximity of other electrodes and not the exact distance between electrodes and modiolar wall. Further the study [27] reports that an increase in distance between SME contacts and the modiolar wall leads to increased impedance, especially in the apical region of the cochlea compared to the basal turn, contrary to existing reports on SME arrays [38,39].

One study focused on probing the effect of modiolar distance on the resistance parameters in an electrode array [31]. The authors reported that the modiolar distance did not have a significant effect on the impedance parameters, despite the large variation in modiolar distance along the electrode array [31]. Possible reasons for this discrepancy include differences in electrode design, scala locations, and cochlear morphology. However, further studies are needed to fully understand the effect of modiolar distance on resistance parameters.

### TIM vs imaging

An interesting argument on the application of TIM in clinical scenario is reduced reliance on the usage of imaging (X-ray, fluoroscopy) measures intraoperatively. This is in line with the hypothesis that TIM can effectively identify electrode malposition, which would reduce the need for routine intraoperative imaging. This would minimize radiation exposure, duration of the surgery and in turn minimize the overall burden on operating theatre. Five studies [24,26,28,33,34] have reported same point of TIM and imaging measurement intraoperatively to check the electrode positioning. While current evidence is not definitive with respect to the replacement of imaging in CI, early reports from the studies within this review are encouraging.

One study has explored evaluating the accuracy of imaging (fluoroscopy) and TIM in detecting TFO in patients implanted with flexible, thin electrodes [28]. Otologists, fellows and residents could detect TFO from TIM heat maps with higher accuracy than with imaging, which further strengthens the arguments for TIM over imaging.

Having considered the disadvantages of imaging, some limitations of TIM are highlighted. Although, particularly in malformed cochleae, routine impedance measures and electrical compound action potentials (ECAP) recordings cannot determine correct positioning, the utility of TIM in these cases are unproven. TIM patterns associated with electrode insertion into the vestibule or semicircular canals have not been reported in the literature. Intraoperative X-ray remains a reliable method for detecting abnormal electrode placement [33]. At this point of time, it is believed in many centres that plain film X-ray has been found to have the greatest impact on surgical decision making and is therefore used as standard of care. Intraoperative X-ray can give additional benefits, such as counting the number of electrodes and measuring angular depth of insertion [33,34].

Attempts have made to categorize hearing pathologies based on TIM measurements [30] in otosclerosis. This could lead to a possible use of TIM in deciding the preoperative electrode array choice and to categorize cochlear anatomy for various predictive models. Further, exploration of TIM with different hearing pathologies such as congenital hearing loss with and without ear malformations may yield better TIM prediction in such populations.

### Overall sensitivity and specificity

From the studies included in the review, TIM has reported high sensitivity and specificity of above 95% [26,29,33]. This suggests the measurement is highly predictive in correctly identifying electrode malposition in normal cochlea. Interestingly, TIM accurately identifies TFOs and buckling more robustly than EE. This could be partly because of nature of voltage spread inside the cochlea, compared to outside of the cochlea. Overall, the predictive value is very high for TFO. One research group [28] has developed an algorithm that was found to be very effective in identification of TFOs. This algorithm has a high positive predictive value (76%), with an incidence of 4% TFOs in SME arrays.

### Limitations

One limitation of the TIM tool is the lack of normative data to guide clinicians in interpreting the measurement. This could be partly due to the accessibility of TIMs to certain clinics/countries. Restricting which centres have access to TIM force clinicians to rely on intraoperative imaging for information regarding the location of the electrode array during surgery. Another possible limitation was the application of TIM in cochlear anomalies including cochlear hypoplasia and incomplete partition of the cochlea. In such cases the TIM presentation may exhibit heterogeneity in nature.

### Future scope of research

TIM is undoubtedly a potentially valuable tool to replace or supplement intraoperative imaging in OT during CI surgery. However, further studies focusing on varying electrode and cochlear characteristics are needed. Further, profiles of TIM measures in abnormal cochleae are needed to establish the positioning of the electrode array in such patients. It is worth studying relationship of the voltage profile of TIM with that of PECAP measures, as these may impact postoperative outcome in CIs. A large-scale data set of TIM collected from patients with normal cochleae would derive a normalized value for TIMs, helping guide the clinicians to clinical decision making. Further implementation of machine learning models and automation of TIM may stratify the pathological classification of patients based on their hearing pathologies.

## Conclusion

TIM use is in the early stages of its clinical adoption. Though TIM appears to have very good potential to identify electrode malposition, the strength of published evidence is at too early a stage to advocate that it be applied in clinical practice as a sole modality. Intraoperative imaging, where necessary, may still be needed to confirm electrode position at this point of time if there are any uncertainties. Combined use of TIM and imaging would be optimal to study further the correlations and reliability of TIM to probe electrode/cochlear interactions.

## Supporting information

S1 File Preferred Reporting Items for Systematic reviews and Meta-Analyses extension for Scoping Reviews (PRISMA-ScR) checklist. (DOCX)

S2 File Example search strategy. (DOCX)

## Abbreviations

CI: Cochlear Implant
ECAP: Electrical Compound Action Potential
EE: Extracochlear Electrodes
NRT: Neural Response Telemetry
PECAP: Panoramic Electrical Compound Action Potential
SCINSEV: Stimulating Current Induced Non-Stimulating Electrode Voltages
SOE: Spread of Excitation
TFO: Tip Fold Over
TIM: Trans Impedance Matrix

## References

[1] Gaylor JM, Raman G, Chung M, et al. Cochlear implantation in adults: A systematic review and meta-analysis. JAMA Otolaryngol Head Neck Surg 2013;139:265–72. doi: 10.1001/jamaoto.2013.1744 23429927

[2] Dorman MF, Natale S, Understanding LLS, Source S. Localization by Cochlear Implant Listeners Using a Pinna-Effect Imitating Microphone and an Adaptive Beamformer. J Am Acad Audiol. 2018;29: 197–205. 29488870 10.3766/jaaa.16126

[3] Kan A, L RY. Binaural hearing with electrical stimulation. Hear Res. 2015;322: 127–137. doi: 10.1016/j.heares.2014.08.005 25193553 PMC4346541

[4] Sousa AF, Miv C, M-C AC. Quality of life and cochlear implant: results in adults with postlingual hearing loss. Braz J Otorhinolaryngol. 2018;84: 494–499. doi: 10.1016/j.bjorl.2017.06.005 28728951 PMC9449166

[5] Ying Y-LM. Lin J, Oghalai JS, W RA. Cochlear implant electrode misplacement: Incidence, evaluation, and management. Laryngoscope. 2013;123: 757–766. doi: 10.1002/lary.23665 23299627 PMC3623683

[6] O’Connell BP, Cakir A, Hunter JB, others. Electrode location and angular insertion depth are predictors of audiologic outcomes in cochlear implantation. Otol Neurotol. 2016;37: 1016–1023. doi: 10.1097/MAO.0000000000001125 27348391 PMC4983244

[7] Dhanasingh A, Jolly C. Review on cochlear implant electrode array tip fold-over and scalar deviation. J Otol. 2019;14: 94–100. doi: 10.1016/j.joto.2019.01.002 31467506 PMC6712287

[8] Wanna GB, Noble JH, Carlson ML, others. Impact of electrode design and surgical approach on scalar location and cochlear implant outcomes. Laryngoscope. 2014;124: S1–7. doi: 10.1002/lary.24728 24764083 PMC4209201

[9] Zanetti D, Nassif N. Redaelli de Zinis LO. Factors Affect residual Hear Preserv cochlear Implant. 2015;35: 433–441. 10.14639/0392-100X-619 PMC4755056 26900250

[10] Ramos-Macias A, De Miguel A R., F-G JC. Mechanisms of electrode fold-over in cochlear implant surgery when using a flexible and slim perimodiolar electrode array. Acta Otolaryngol. 2017;137: 1129–1135. doi: 10.1080/00016489.2016.1271449 28784019

[11] De Rijk SR, Tam YC, Carlyon RP, Bance ML. Detection of extracochlear electrodes in cochlear implants with electric field imaging/transimpedance measurements: A human Cadaver study. Ear Hear. Publ online January. 2020;7. doi: 10.1097/AUD.0000000000000837 31923041 PMC7115972

[12] Zuniga MG, Rivas A, Hedley-Williams A, others. Tip fold-over in cochlear implantation: case series. Otol Neurotol. 2017;38: 199–206. doi: 10.1097/MAO.0000000000001283 27918363 PMC5584995

[13] Holder JT, Kessler DM, Noble JH, Gifford RH, L RF. Prevalence of extracochlear electrodes: Computerized tomography scans, cochlear implant maps, and operative reports. Otol Neurotol. 2018;39: 325–331. doi: 10.1097/MAO.0000000000001818 29738386 PMC7197293

[14] Brown KD, Connell SS, Balkany TJ, Eshraghi AE, Telischi FF, A SA. Incidence and indications for revision cochlear implant surgery in adults and children. Laryngoscope. 2009;119: 152–157. doi: 10.1002/lary.20012 19117299

[15] Mittmann P, Lauer G, Ernst A, others. Electrophysiological detection of electrode fold-over in perimodiolar cochlear implant electrode arrays: a multi-center study case series. Eur Arch Oto-rhino-laryngol. 2020;277: 31–35. doi: 10.1007/s00405-019-05653-9 31552525

[16] Swaddiwudhipong N, Jiang C, Landry TG, Bance M. Investigating the Electrical Properties of Different Cochlear Implants. Otol Neurotol. 2021;42: 59–67. doi: 10.1097/MAO.0000000000002861 32941302 PMC7737872

[17] Ramos-Macias A, Riol Sancho D. Falcón-González J. C; Pavone J; Rodriguez Herrera L; Borkoski Barreiro S; et al, Assess Place Cochlear Slim Perimodiolar Electrode Array by Trans Impedance Matrix Anal A Temporal Bone Study. 2022;11: 3930. 10.3390/jcm11143930 PMC9317462 35887693

[18] Vanpoucke FJ, Zarowski AJ, P SA. Identification of the impedance model of an implanted cochlear prosthesis from intracochlear potential measurements. IEEE Trans Biomed Eng. 2004;51: 2174–2183. doi: 10.1109/TBME.2004.836518 15605865

[19] Vanpoucke FJ, Boermans PP, F JH. Assessing the placement of a cochlear electrode array by multidimensional scaling. IEEE Trans Biomed Eng. 2012;59: 307–310. doi: 10.1109/TBME.2011.2173198 22042122

[20] Levac D, Colquhoun H, studies: advancing the methodology. Implement science IS. 2010;5: 69. 20854677 10.1186/1748-5908-5-69 PMC2954944

[21] Tricco AC, Lillie E, Zarin W, O’Brien KK, Colquhoun H, Levac D, et al. PRISMA Extension for Scoping Reviews (PRISMA-ScR): Checklist and ExplanationThe PRISMA-ScR Statement. Ann Intern Med. 2018; 169(7); pp. 467–473. 30178033 10.7326/M18-0850

[22] Cumpston M, Li T, Page MJ, others. Updated guidance for trusted systematic reviews: a new edition of the Cochrane Handbook for Systematic Reviews of Interventions. Cochrane Database Syst Rev. 2019;10. doi: 10.1002/14651858.ED000142 31643080 PMC10284251

[23] Downs SH, Black N. The feasibility of creating a checklist for the assessment of the methodological quality both of randomized and non-randomized studies of health care interventions J. Epidemiol Community Heal. 1998;52: 377–384. 10.1136/jech.52.6.377 PMC1756728 9764259

[24] Klabbers TM, Huinck WJ, Heutink F, Verbist BM, (tim) MEAMTM. Measurement for the Detection of Intraoperative Electrode Tip Foldover Using the Slim Modiolar Electrode: A Proof of Concept Study. Otol Neurotol. 2021;42: 2. 32941298 10.1097/MAO.0000000000002875

[25] Soderqvist S, Lamminmaki S, Aarnisalo A, Hirvonen T, Sinkkonen ST, S VI. transimpedance and spread of excitation profile correlations with a lateral-wall cochlear implant electrode array. Hear Res. 2021;405: 5. 10.1016/j.heares.2021.108235 33901994

[26] Hans S, Arweiler-Harbeck D, Kaster F, others. Transimpedance Matrix Measurements Reliably Detect Electrode Tip Fold-over in Cochlear Implantation. Otol Neurotol. 2021;42: 10. 34766947 10.1097/MAO.0000000000003334

[27] Ramos-de-Miguel A, Falcón-González JC, Ramos-Macias A. Analysis of Neural Interface When Using Modiolar Electrode Stimulation. Radiol Eval Trans-Impedance Matrix Anal Eff List Effort Cochlear Implant. 2021;10: 17. 10.3390/jcm10173962 PMC8432261 34501410

[28] Klabbers TM, Huinck WJ, M EAM. Comparison Between Transimpedance Matrix (TIM) Measurement and X-ray Fluoroscopy for Intraoperative Electrode Array Tip Fold-Over Detection. Otol Neurotol. 2021;42: 10. 34238897 10.1097/MAO.0000000000003290

[29] Hoppe U, Brademann G, Stöver T, others. Evaluation of a Transimpedance Matrix Algorithm to Detect Anomalous Cochlear Implant Electrode Position. Audiol Neurootol. 2022;27: 347–355. doi: 10.1159/000523784 35306487

[30] Vozzi A, Ronca V, Malerba P, others. An innovative method for trans-impedance matrix interpretation in hearing pathologies discrimination. Med Eng Phys. 2022;102: 1. doi: 10.1016/j.medengphy.2022.103771 35346431

[31] Leblans M, Sismono F, Vanpoucke F, others. Novel Impedance Measures as Biomarker for Intracochlear Fibrosis. Hear Res. 2022;426: 3. doi: 10.1016/j.heares.2022.108563 35794046

[32] de Rijk SR, Hammond-Kenny A, Tam YC, others. Detection of Extracochlear Electrodes Using Stimulation-Current- Induced Non-Stimulating Electrode Voltage Recordings With Different Electrode Designs. Otol Neurotol. 2022;43: 5. doi: 10.1097/MAO.0000000000003512 35617005

[33] Kay-Rivest E, McMenomey SO, Jethanamest D, others. Predictive Value of Transimpedance Matrix Measurements to Detect Electrode Tip Foldover. Otol Neurotol. 2022;43: 1027–1032. doi: 10.1097/MAO.0000000000003667 36040040

[34] Cheung LL, Kong J, Chu PY, Sanli H, Walton J, B CS. Misplaced Cochlear Implant Electrodes Outside the Cochlea: A Literature Review and Presentation of Radiological and Electrophysiological Findings. Otol Neurotol. 2022;43: 567–579. doi: 10.1097/MAO.0000000000003523 35261380

[35] Coombs A, Clamp PJ, Armstrong S, Robinson PJ, Hajioff D. The role of post-operative imaging in cochlear implant surgery: A review of 220 adult cases. Cochlear Implant Int. 2014;15: 264–271. doi: 10.1179/1754762814Y.0000000071 24679147

[36] Goh X, Harvey L, Axon PR, others. Frequency of electrode migration after cochlear implantation in the early postoperative period. What are Assoc risk factors? [published online ahead print. 2023; 13. 10.1111/coa.14062 37051731

[37] Garcia C, Goehring T, Cosentino S, et al. The Panoramic ECAP Method: Estimating Patient-Specific Patterns of Current Spread and Neural Health in Cochlear Implant Users. J Assoc Res Otolaryngol. 2021;22:567–589. doi: 10.1007/s10162-021-00795-2 33891218 PMC8476702

[38] Garaycochea O, Manrique-Huarte R, Lazaro C, et al. Comparative study of two different perimodiolar and a straight cochlear implant electrode array: surgical and audiological outcomes. Eur Arch Otorhinolaryngol. 2020;277: 69–76. doi: 10.1007/s00405-019-05680-6 31637478

[39] Kim Y, Kim Y, Kim YS, Lee SY, Choi BY. Tight modiolar proximity and feasibility of slim modiolar cochlear implant electrode array insertion in diverse etiologies of hearing loss. Eur Arch Otorhinolaryngol. 2022;279:3899–3909. doi: 10.1007/s00405-021-07150-4 34718854

