# Supplementary material for: A scoping review on the clinical effectiveness of Trans-Impedance Matrix (TIM) measurements in detecting extracochlear electrodes and tip fold overs in Cochlear Ltd devices
Source: PLoS One. 2024 Mar 7;19(3):e0299597. doi: 10.1371/journal.pone.0299597 (PMC10919746; doi:10.1371/journal.pone.0299597)
Supplement: S2 File — (DOCX) [file pone.0299597.s002.docx]

# **S2. Example search strategy: Medline via Ovid**

((cochlear or auditory) adj5 (implant* or prosthe*) or "round window" or "promontory" or "auditory nerve*" or "cochlear nerve*").ti,ab,kw. OR Cochlear Implants/ or Cochlear Implantation/ or Round Window, Ear/ or Cochlear Nerve/

AND

(TIM or impedance or resistance* or "trans-impedance matrix").ti,ab,kw. OR electric impedance/

AND

("Tip fold over" OR "Extra cochlear electrode*" OR "electrodes outside the cochlea" OR "bending of the electrodes" OR "bending of electrodes").ti,ab,kw.
